# Dipeptidyl Peptidase-4 inhibitors use in type II diabetic patients in a tertiary hospital

**DOI:** 10.1186/s40545-020-00238-y

**Published:** 2020-06-18

**Authors:** Woh Yon Mak, Jivanraj R. Nagarajah, Hannah Abdul Halim, Anitha Ramadas, Zulsairi Mohd Pauzi, Lay Ting Pee, Nirmala Jagan

**Affiliations:** Department of Pharmacy, Hospital Kuala Lumpur, Kuala Lumpur, Malaysia

**Keywords:** Dipeptidyl peptidase 4 inhibitors (DPP4-i), HbA1c, Type II diabetes mellitus (T2DM), Southeast Asia

## Abstract

**Background:**

In Malaysia, for more than a decade, dipeptidyl peptidase-4 inhibitors (DPP-4i) are among the oral antidiabetic medications used as monotherapy or in combination to manage type II diabetes mellitus (T2DM). These medications are known for the efficacy in glycated haemoglobin (HbA1c) reduction and weight neutral effect with minimal hypoglycaemia occurrence. This study aimed to identify the outcomes of DPP-4i use in one of the largest tertiary public hospital in Southeast Asia.

**Methods:**

This is a retrospective cross sectional study conducted in 2016, where stratified sampling method was used. Patients with T2DM treated with available DPP-4i; namely Linagliptin, Saxagliptin, Sitagliptin and Vildagliptin, for at least 3 months were identified from the pharmacy record. Medical records from Physician Clinic in Hospital Kuala Lumpur (HKL) were reviewed. Data on demographic, anthropometric, antidiabetic treatment modalities, laboratory and documented outcomes were collected. Outcomes endpoints which include changes in HbA1c, fasting blood glucose (FBG), and body weight were recorded and analysed. Adverse drug reactions (ADR) documented were also reported.

**Results and discussion:**

A total of one hundred and five patients were recruited. The patients were 49.5% men (*n* = 52), with a mean age of 57 years, mean HbA1c of 8.5% (69 mmol/mol) and mean BMI of 29.5 kg/m^2^. At least 50% of the patients had T2DM for more than 10 years and more than two third of these patients had both T2DM and hypertension. Thirty nine patients were on Vildagliptin, 32 on Sitagliptin, 26 on Saxagliptin and the remaining on Linagliptin. The most commonly prescribed DPP-4i were Vildagliptin and Sitagliptin. Majority of the patients (90.4%) were prescribed with Metformin, with 62.8% of patients on fixed-dose combination, and the remaining on add-on Metformin therapy. Use of DPP-4i as an adjunct was associated with a mean reduction of 0.9% (9 mmol/mol) in HbA1c (*p* < 0.0001) and 1.15 mmol/L (19.82 mg/dL) in FBG (*p* = 0.001) without significant weight changes (*p* = 0.745). Sitagliptin had the highest reduction in HbA1c (1.66%,19 mmol/mol; *p*-value< 0.0001). Twelve ADRs were reported with the highest report on gastrointestinal intolerance (*n* = 7). None of the ADR reported caused any significant harm to the patients.

**Conclusion:**

Overall, use of these DPP-4i as an adjunct antidiabetic was associated with reduction in HbA1c.

## Background

### Literature review

Globally, International Diabetes Federation (IDF) [1] reported diabetes prevalence figure of 1 in 11 adults. The figure will increase to 1 in 10 adults by 2040. According to IDF, 12% of the global health expenditure of approximately 727 billion USD was spent in 2017 in managing patients with diabetes and the figure is expected to increase further in future. Malaysia has one of the highest and most rapidly increasing prevalence of diabetes in the Western Pacific region. The prevalence of diabetes in Malaysia reported in National Health & Morbidity Survey 2015 (NHMS) [2] had increased from 8.3% in year 1996 to 17.5% in year 2015. In terms of diabetes control, majority of Type 2 Diabetes Mellitus (T2DM) patients in our country failed to achieve their individual glycaemic targets, with only 12.7% of T2DM patients in tertiary hospitals able to attain their glycaemic targets [3, 4].

Dipeptidyl peptidase-4 inhibitors (DPP-4i) are agents that increase glucagon like peptide-1 (GLP-1) and gastric inhibitory polypeptide (GIP) levels, which inhibit glucagon release leading to increased insulin secretion and thereby reduce blood glucose levels [5]. DDP-4 is a protease involved in GLP-1 inactivation. By inhibiting the enzyme, DPP-4i prolong and enhance the activity of GLP-1. GLP-1 exerts its main effects by stimulating glucose dependent insulin release, slowing gastric emptying, reducing food intake, and decreasing postprandial glucagon excretion [6, 7].

DPP-4i have been incorporated into numerous guidelines available for the management of patients with T2DM. American Diabetes Association (ADA) and European Association for the Study of Diabetes (EASD) positioned DPP-4i alongside with sulfonylureas, thiazolidinediones, GLP-1 agonists and insulin, as a second-line add-on to metformin in their general recommendation [8].

According to the National Institute for Health and Clinical Excellence (NICE) guideline on newer agents for blood glucose control in T2DM published in 2009, DPP-4i can be considered as a second-line therapy to first-line metformin if there is a significant risk of hypoglycaemia or if a sulfonylurea is contraindicated or not tolerated [9].

According to the Malaysian Clinical Practice Guideline on the Management of T2DM 2015, DPP-4i use is recommended early in patients with HbA1c of 6.5% (48 mmol/mol) and above as monotherapy or in combination with other antidiabetic agents [10]. DPP-4i that are available in Malaysia include Sitagliptin, Saxagliptin, Vildagliptin, Alogliptin and Linagliptin [11]. Although there are noteworthy pharmacokinetic and pharmacodynamic differences among various types of DPP-4i, there is no clear local or national guideline stating the selection criteria of patients to be started on a certain type of DPP-4i [12]. Huri HZ et al. did a retrospective review to analyse the utilization patterns of DPP-4 inhibitors and identified age, concomitant use of beta blockers and aspirin as factors associated with the use of DPP-4i among Malaysia University Malaya Medical Centre (UMMC) patients [13]. However, the study was only limited to Sitagliptin and Vildagliptin.

Currently, DPP-4i are not widely used in Malaysia public healthcare institutions as compared to private healthcare settings [14]. Hospital Kuala Lumpur (HKL) is the largest public tertiary hospital in Southeast Asia with about 11,000 patients actively seeking treatment in its Physician Clinic. HKL physicians have access to four types of DPP-4i, namely Sitagliptin, Saxagliptin, Vildagliptin and Linagliptin. This study aimed to identify the utilization pattern of DPP-4i in HKL and to determine clinical outcomes of patients prescribed with DPP-4i, mainly glycated haemoglobin (HbA1c), fasting blood glucose (FBG) and any occurrence of adverse event.

## Methods

### Study design

This is a retrospective, cross sectional study involving patients under HKL Physician Clinic follow up. Patients included in this study were T2DM patients aged 18 years and above whom were taking any of the available DPP-4i, either as single pill or combination pill, for a minimum of 3 months within year 2011 to 2016. Pregnant patients, patients with psychiatric illness on anti-psychotics, end stage renal dysfunction on regular dialysis were excluded from our study.

### Study population

Study subjects were identified through the patient list on DPP-4i in the year 2016 from the pharmacy record using convenient stratified sampling method. The total minimum sample size which gives 80% study power is 95, calculated using Epi Info™ Software version 7.1.5.2 (CDC), assuming expected proportion of patients on DPP-4i of 9.5% and confidence limit of 5% [15]. From a total of 385 patients who were on DPP-4i, number of patients who fulfilled the inclusion criteria and recruited was 105.

### Data collection

Patients’ records were retrieved from the physician clinic and data collection was conducted using a structured data collection form (Appendix 1). Data collected include types of DPP-4i commonly prescribed, demographic parameters of patients on DPP-4i, anthropometric, antidiabetic treatment modalities, laboratory data and self-reported outcomes. Changes in HbA1c, fasting blood glucose (FBG) and body weight were recorded and analysed. Adverse drug reactions (ADR) documented were also reported.

### Statistical analysis and data interpretation

The demographic, medication details and clinical parameters of the study were analysed using descriptive statistics, reported in frequency and percentage. Continuous data were reported as mean ± standard deviation (SD). Paired T-test was conducted to compare the mean difference of continuous variables pre- and post DPP-4i while Independent T-test and Analysis of variance (ANOVA) were conducted to compare the HbA1c difference between the groups. Post-hoc analysis (Least Significant Differences, LSD) was conducted for significant ANOVA outcomes. Meanwhile, the proportion or categorical variables were compared using Pearson chi-square test. All statistical analyses were conducted using IBM SPSS Statistics version 20.0 where *p*-value of < 0.05 was considered statistically significant.

## Results

### Demographic description

A total of 105 patients, 52 (49.5%) men and 53 (50.5%) women were included in this study. The baseline demographic and clinical characteristics of the patients are described in Table 1 and Table 2, respectively. Mean age of the patients was 57.0 ± 12.1 years, almost half (43.8%) of them were above 60 years. Two-third of the patients (60%) were obese (body mass index ≥27.5 kg/m^2^) and about one-third of them (36%) were overweight (body mass index 23–27.4 kg/m^2^), with mean body mass index (BMI) of 29.5 ± 4.6 kg/m^2^.

**Table 1:** Patient’s baseline demographic characteristics, *N* = 105

Parameters/characteristics	n (%)
Age (years old)^a^	57.0 ± 12.1
< 60	59 (56.2)
≥ 60	46 (43.8)
Gender	
Male	52 (49.5)
Female	53 (50.5)
Race	
Malay	53 (50.5)
Chinese	19 (18.0)
Indian	32 (30.5)
Others	1 (1.0)
Smoking	
Yes	2 (1.9)
No	93 (88.6)
Ex-smoker	10 (9.5)
Alcohol consumption	
Yes	1 (1.0)
No	101 (96.1)
Ex-consumer	3 (2.9)
Weight (kg)^a^	78.1 ± 14.5
Height (m)^a^	1.62 ± 0.08
BMI (kg/m^2^)^a, b^	29.5 ± 4.6
< 18.5 (Underweight)	0 (0.0)
18.5–22.9 (Normal)	4 (3.8)
23.0–27.4 (Overweight)	38 (36.2)
≥ 27.5 (Obese)	63 (60.0)
Waist circumference (cm)^a^	
Male	99.7 ± 11.5
Female	94.9 ± 11.8

^a^Continuous variable reported as mean ± SD

^b^Classification of weight by BMI adapted from Malaysian Clinical Practice Guideline on the Management of T2DM 2015 [10]

**Table 2:** Patient’s baseline clinical characteristics, *N* = 105

Parameters/characteristics	n (%)
Duration of T2DM (years)^a^	11.5 ± 8.5
≤ 10	53 (50.5)
11–20	39 (37.1)
21–30	8 (7.6)
> 30	5 (4.8)
Comorbidities	
Hypertension	74 (70.5)
Dyslipidaemia	50 (47.6)
Ischaemic heart disease	12 (11.4)
Cerebrovascular accident	2 (1.9)
Renal impairment	13 (12.4)
Liver impairment	9 (8.6)
Heart failure	5 (4.8)
Diabetic complications	
Neuropathy	9 (8.6)
Nephropathy	5 (4.8)
Retinopathy	12 (11.4)
All of the above	2 (1.9)
HbA1c (%)^a^	8.5 ± 1.8
< 6.5	8 (7.6)
6.5–9.9	76 (72.4)
≥ 10	21 (20.0)
FBG (mmol/L)^a^	9.1 ± 3.8
ALT (μmol/L)^a^	31.0 ± 0.2
TC (mmol/L)^a^	5.0 ± 4.0
TG (mmol/L)^a^	1.9 ± 1.3
LDL (mmol/L)^a^	2.6 ± 0.9
HDL (mmol/L)^a^	1.2 ± 0.4

^a^Continuous variable reported as mean ± SD

Mean HbA1c before initiation of DPP-4i was 8.5 ± 1.8%, in which about 72.4% of the patients had HbA1c ranging from 6.6 to 9.9%. Mean baseline FBG was 9.1 ± 3.8 mmol/L (163.8 ± 68.47 mg/dL). Mean duration of T2DM was 11.5 ± 8.5 years and about half of the patients (52%) had long-standing T2DM (> 10 years). Hypertension (~ 70%) and dyslipidaemia (~ 50%) were the main comorbidities followed by ischaemic heart disease (~ 11%), renal impairment (~ 9%), liver impairment (~ 9%) and heart failure (~ 5%). Approximately 30% of the patients had diabetic complications prior to DPP-4i treatment, 12% retinopathy, 9% neuropathy and 5% nephropathy.

### Patterns of DPP-4i use

The patterns of DPP-4i use are described in Table 3. The usage of Vildagliptin, Sitagliptin and Saxagliptin was comparable, with 37% (*n* = 39) of patients on Vildagliptin, 30.5% (*n* = 32) of them on Sitagliptin and 24.8% (*n* = 26) of the patients were on Saxagliptin. The lowest DPP-4i usage was Linagliptin, 7.6% (*n* = 8). Two third of the patients (62.8%, *n* = 66) were on fixed-dose combination (FDC) therapy. Only one patient was on DPP4-i monotherapy of Linagliptin alone. The mean duration of exposure to DPP-4i was 29.7 months. Almost all DPP-4i were administered with Metformin (90.5%) either in fixed-dose combination (62.9%) or add on tablets (27.6%). Other concomitant antidiabetics include insulin (56.2%), sulphonylurea (26.7%) and Acarbose (2.9%).

**Table 3:** Patterns of DPP-4i use, *N* = 105

Parameters/characteristics	n (%)
Types of DPP-4i	
Vildagliptin	39 (37.1)
Sitagliptin	32 (30.5)
Saxagliptin	26 (24.8)
Linagliptin	8 (7.6)
Duration of use (months) ^a^	29.7 ± 19.8
< 24	46 (43.8)
≥ 24	59 (56.2)
Fixed-dose combination (FDC) tablets	
Vildagliptin/Metformin	27 (25.7)
Sitagliptin/Metformin	21 (20.0)
Saxagliptin/Metformin	18 (17.1)
Metformin	
FDC	66 (62.9)
Add on	29 (27.6)
Total	95 (90.5)
Other concurrent antidiabetics	
Insulin	59 (56.2)
Gliclazide IR/MR	27 (25.7)
Glibenclamide	1 (1.0)
Acarbose	3 (2.9)
None	28 (26.7)
Types of insulin therapy	
Basal bolus	28 (26.7)
Premixed	15 (14.3)
Bolus only	15 (14.3)
Basal only	1 (1.0)
Adherence	
Good	58 (55.2)
Poor	13 (12.4)
Not documented	34 (32.4)

^a^Continuous variable reported as mean ± SD

### Outcomes of DPP-4 inhibitors usage

The outcomes of DPP-4i are described in Tables 4, 5, 6, 7, 8 and 9. Use of DPP-4 inhibitors showed a mean reduction in HbA1c of 0.9% and reduction in FBG of 1.15 mmol/L (19.82 mg/dL). The largest reduction in HbA1c were noted among patients on Sitagliptin (mean difference = 1.66%, *p* < 0.0001) and Vildagliptin (mean difference = 0.68%, *p* = 0.011). The post hoc analysis showed significantly higher reduction in HbA1c in patients on Sitagliptin compared to other DPP-4i, where Sitagliptin versus Vildagliptin (mean difference = 0.99%, *p* = 0.010) and Sitagliptin versus Saxagliptin (mean difference = 1.33%, *p* = 0.002). Subjects on oral antidiabetics without insulin therapy were analysed as per Table 5b. There was significant change in HbA1c pre and post DPP-4i use even without concurrent insulin therapy (*p* < 0.0001). Larger reduction in HbA1c were observed among those on Sitagliptin and Vildagliptin and there was no significant difference in the HbA1c drop between the types of DPP-4i used in patients who were not on insulin.

**Table 4:** Outcomes of DPP-4i use, N = 105

Parameters	Pre DPP-4i use	Post DPP-4i use	Mean difference (95% CI)	p value^**£**^
HbA1c (%)	8.5 ± 1.8	7.6 ± 1.7	− 0.90 (− 1.22, − 0.58)	< 0.0001*
FBG (mmol/L)	9.2 ± 3.8	8.0 ± 3.2	−1.15 (− 1.80, − 0.51)	0.001*
Weight (kg)	78.1 ± 14.5	78.1 ± 14.4	0.08 (− 0.41,0.57)	0.745
BMI (kg/m^2^)	29.5 ± 4.6	30.0 ± 7.0	0.50 (− 0.51,1.51)	0.332

^£^Paired T-test conducted. *p* value is significant at< 0.05

Values reported as mean ± SD.

**Table 5:** HbA1c pattern, *N* = 105

HbA1c	Pre DPP-4i use n (%)	Post DPP-4i use n (%)
**< 6.5%**	8 (7.6)	21 (20.0)
**6.5–9.9%**	76 (72.4)	70 (66.7)
**≥10%**	21 (20.0)	14 (13.3)

χ^2^ = 52.56, df = 4, *p* value < 0.0001 (significant)

**Table 6:** HbA1c pattern, *N* = 47 (patients without insulin)

HbA1c	Pre DPP-4i use n (%)	Post DPP-4i use n (%)
**< 6.5%**	5 (10.6))	14 (29.8)
**6.5–9.9%**	37 (78.8)	32 (68.1)
**≥10%**	5 (10.6)	1 (2.1)

χ^2^ = 43.57, df = 2, *p* value < 0.0001 (significant).

**Table 7:** Change in HbA1c with DPP-4i use, *N* = 105

	Pre DPP-4i use	Post DPP-4i use	Mean difference ± 95% CI	***P*** value^**£**^	***P*** value^**¥**^
Age (years old)					
< 60	8.7 ± 2.0	7.8 ± 1.7	0.89 (0.44,1.34)	< 0.0001*	0.963
≥ 60	8.3 ± 1.5	7.4 ± 1.6	0.91 (0.45,1.36)	< 0.0001*	
Type of DPP-4i					
Vildagliptin	8.1 ± 1.5	7.4 ± 1.6	0.68 (0.21, 1.14)	0.006*	0.011^¢^
Sitagliptin	9.2 ± 2.2	7.5 ± 1.8	1.66 (0.95, 2.37)	< 0.0001*	
Saxagliptin	8.4 ± 1.4	8.0 ± 1.5	0.33 (−0.03, 0.67)	0.052	
Linagliptin	8.8 ± 2.1	8.1 ± 2.1	0.76(−1.12, 2.64)	0.370	
Gender					
Male	8.5 ± 1.8	7.4 ± 1.5	1.13 (0.59, 1.66)	< 0.0001*	0.162
Female	8.6 ± 1.8	7.9 ± 1.8	0.68 (0.31, 1.04)	< 0.0001*	
Therapy					
FDC	8.6 ± 1.8	7.6 ± 1.7	0.99 (0.60, 1.38)	< 0.0001*	0.455
Single pill	8.5 ± 1.8	7.8 ± 1.7	0.74 (0.18, 1.30)	0.011*	
Insulin					
Yes	9.0 ± 2.0	8.2 ± 1.9	0.81 (0.37, 1.25)	< 0.0001*	0.554
No	8.0 ± 1.5	7.0 ± 1.1	1.00 (0.53, 1.48)	< 0.0001*	
Sulphonylurea					
Yes	8.2 ± 1.1	7.2 ± 1.1	0.96 (0.36, 1.56)	0.003*	0.824
No	8.7 ± 2.0	7.8 ± 1.8	0.88 (0.49, 1.26)	< 0.0001*	

^£^Paired T-test conducted to compare pre and post DPP-4i HbA1c

^¥^Independent T-test and ANOVA conducted to compare the HbA1c difference between the groups

^¢^Post-hoc analysis (LSD) conducted for types of DPP-4i

Values reported as mean ± SD

*p value is significant at< 0.05

**Table 8:** Change in HbA1c with DPP-4i use, *N* = 47 (patients without insulin)

Type of DPP-4i	n (%)	Pre DPP-4i use	Post DPP-4i use	Mean difference (95% CI)	***P*** value^**£**^	***P*** value^**¥**^
Vildagliptin	20 (42.6)	7.7 ± 0.9	6.7 ± 0.8	1.03 (0.37, 1.68)	0.004*	0.104^¢^
Sitagliptin	13 (27.7)	8.8 ± 2.1	7.1 ± 1.4	1.69 (0.25, 3.13)	0.025*	
Saxagliptin	13 (27.7)	7.8 ± 1.3	7.4 ± 1.1	0.32(−0.06, 0.71)	0.094	
Linagliptin	1 (2.1)					

^£^Paired T-test conducted to compare pre and post DPP-4i HbA1c

^¥^ANOVA conducted to compare the HbA1c difference between the groups

Values reported as mean ± SD

*p value is significant at< 0.05

Linagliptin omitted from analysis as *n* = 1

**Table 9:** ADR related to DPP-4i use, *N* = 105

Parameters/characteristics		n (%)
ADR occurrence	Saxagliptin	6
	Sitagliptin	3
	Linagliptin	0
	Vildagliptin	3
Types of ADR	Gastrointestinal intolerance	7 (6.7)
	Dizziness	3 (2.9)
	Hypoglycaemia	1 (1.0)
	Leg oedema	1 (1.0)
Action taken when ADR occurs	Change to different DPP-4i	6 (50.0)
	Continue the same DPP-4i	5 (42.7)
	Dose reduction	1 (8.3)
	Discontinuation of DPP-4i	0 (0.0)
Choice of new DPP-4i	Saxagliptin/Metformin	4 (50.0)
	Saxagliptin	1 (33.3)
	Linagliptin	1 (16.7)

A total of 12 cases of adverse drug reactions (ADR) were reported among the study population (12%), six reports from Saxagliptin, three from Sitagliptin and another three reports from Vildagliptin. Most common ADR was gastrointestinal intolerance (6.7%, *n* = 7), followed by dizziness (3%, *n* = 3), hypoglycaemia (1%, *n* = 1) and leg oedema (1%, *n* = 1).

Seven patients experienced gastrointestinal intolerance, three patients were on Saxagliptin/Metformin, two patients on Vildagliptin/Metformin and another two patients were on Sitagliptin/Metformin. One of them was switched to Linagliptin monotherapy as patient developed gastrointestinal intolerance due to Metformin. One patient on Vildagliptin/Metformin and two patients on Sitagliptin/Metformin were switched to Saxagliptin single pill without Metformin. The remaining three patients had their current FDC of DPP-4i continued.

One patient on Vildagliptin/Metformin complained of dizziness and was switched to Saxagliptin/Metformin. Meanwhile, another two cases of dizziness reported by patients on Saxagliptin/Metformin were continued on the same therapy. One case of bilateral leg oedema was reported with Sitagliptin/Metformin and was switched to Saxagliptin/Metformin. Only one case of hypoglycaemia was reported but that particular patient was on Saxagliptin/Metformin, Gliclazide and basal insulin concurrently.

No significant weight change was observed pre and post DPP4i use (mean difference + 0.08 kg).

## Discussion

Malaysian Clinical Practice Guideline on the Management of T2DM has included DPP-4i as an alternative or addition to Metformin in managing T2DM patients with HbA1c of 6.5 to 10% [10]. Majority of the patients in this study had HbA1c in the range of 6.6 to 9.9% before initiation of DPP-4i.

DPP-4i can be started regardless of how long patient have had diabetes as its efficacy is not influenced by the duration of T2DM [16–21]. Time of starting DPP-4i to the duration of diabetes is less than 10 years in half of the patients studied. The average age of patients on DPP4i in this study was 57 years with two third of the patients being either overweight (BMI 23–27.4 kg/m^2^) or obese (BMI ≥27.5 kg/m^2^). These findings are similar to the data published in the National Diabetes Registry Report [22] on Malaysian T2DM population, where mean age was 59.7 years and average BMI was 27.4 kg/m^2^. In this study, number of T2DM patients was the highest among Malays followed by Indians and Chinese, which again coincides with the data reported in Malaysia National Diabetes Registry Report [22].

Hypertension was the most common comorbidity in our patient population, followed by dyslipidemia and ischaemic heart disease. Huri HZ et al. [13] reported similar results in their study population for hypertension and dyslipidemia while Mafauzy [23] found that hypertension was the most prevalent comorbid condition among diabetic patients from 49 private clinics in Malaysia.

All available DPP-4i were prescribed equally except Linagliptin, which was the least prescribed. FDC pill of DPP-4i with Metformin were preferred compared to single pill DPP4-I as FDC pills can reduce patients’ overall pill burden. DPP-4i are preferred over the traditional choice of sulphonylurea as second line treatment as DPP-4i have low risk of causing hypoglycaemia especially in elderly patients (46% of the study population) [10]. Patients who received FDC pill of DPP4-i and Metformin had significantly larger HbA1c reduction compared to the group receiving non FDC. This is probably due to better compliance towards FDC attributed to the lesser pill burden.

This study had shown significant reduction in HbA1c and FBG post DPP-4i as an adjunct therapy, where there was a drop in HbA1c to below 6.5% post DPP-4i therapy in about a quarter of the study population. On the contrary, studies in other countries to date mainly assessed effectiveness of DPP4-i as monotherapy alone or DPP4-i individual agent compared to other single oral antidiabetic as monotherapy, which resulted an average HbA1c reduction of 0.5 to 0.8% [16–21].

Present study showed that both Sitagliptin and Vildagliptin use notably had larger HbA1c reduction post therapy. The largest HbA1c drop was observed in the group of patients using Sitagliptin. This outcome might be due to the predominantly higher baseline HbA1c in patients on Sitagliptin. Moreover, almost a quarter of the study population had baseline HbA1c of 10% and above, which is considerably higher compared to the baseline HbA1c set as recruitment criteria in other similar studies (7.5–8.5%) [16–21]. We found similar reduction in HbA1c among patients on DPP4-i even without concurrent insulin. A separate analysis is necessary to eliminate the possible confounding effect by concurrent insulin therapy as insulin is reported to be able to reduce HbA1c of more than 1.5% [10].

## Limitations

Several limitations were identified in this study. HbA1c outcomes were obtained at varied duration of DPP4-i use as HbA1c investigation was performed at different time period from the time of DPP4-i initiation for each patient. In addition, more than one third of the patients’ medication adherence were not assessed and documented in this study. Thus the impact of medication adherence towards treatment outcome cannot be determined.

## Conclusion

In conclusion, this study showed that use of DPP-4i as an adjunct was associated with a significant reduction in patients’ HbA1c and FBG without significant weight change. Sitagliptin showed the greatest HbA1c reduction. DPP-4i were well tolerated with no significant reported adverse drug reaction.

## Future direction

This study can be extrapolated prospectively to investigate the factors associated with utilization of DPP4-i. Furthermore, results can be refined by categorizing patients according to concomitant antidiabetic agents used and whether dosages of each agent are optimized.

## Data Availability

All data generated or analysed during this study were included in this article.

## Abbreviations

ADR: Adverse drug reactions
ALT: Alanine transaminase
ANOVA: Analysis of variance
BMI: Body mass index
DPP-4i: Dipeptidyl peptidase-4 inhibitors
FBG: Fasting blood glucose
FDC: Fixed-dose combination
GLP-1: Glucagon like peptide-1
HbA1c: Glycated haemoglobin
HDL: High-density lipoprotein
IR: Immediate release
LDL: Low-density lipoprotein
LSD: Least Significant Differences
MR: Modified release
SD: Standard deviation
T2DM: Type II diabetes mellitus
TC: Total cholesterol
TG: Triglyceride
